# Low-calorie diets are effective for weight loss in patients undergoing benign upper gastrointestinal surgery: a systematic review and meta-analysis

**DOI:** 10.1007/s00464-024-11016-1

**Published:** 2024-07-08

**Authors:** Nibir Chowdhury, Syarafina Hasnan, Shahid Ullah, Sarah K. Thompson

**Affiliations:** 1 College of Medicine & Public Health, Flinders University, Bedford Park, SA Australia; 2 Adelaide Gastrointestinal Specialists, Eastwood, SA Australia; 3 Flinders Medical Centre, Rm 5E221.3, Bedford Park, SA 5042 Australia

**Keywords:** Low-calorie diet, Very low-calorie diet, Weight loss, Liver volume, Bariatric surgery

## Abstract

**Background:**

Obesity may increase surgical complexity in patients undergoing abdominal surgery by limiting visualization and increasing the risk of peri-operative complications. A preoperative reduction in weight and liver volume may improve surgical outcomes. The aim of our study was to evaluate the efficacy of a low-calorie diet (LCD) versus a very low-calorie diet (VLCD) in reducing weight and liver volume prior to laparoscopic surgery.

**Methods:**

A systematic search was conducted using the following inclusion criteria: obese patients undergoing preoperative weight loss using a VLCD or LCD, evaluation of liver volume reduction, and the use of an imaging modality before and after the diet.

**Results:**

A total of 814 patients from 21 different studies were included in this systematic review and meta-analysis, with 544 female patients (66.8%) and a mean age range between 24 and 54 years old. There was a total mean weight loss of 6.42% and mean liver volume reduction of 16.7%. Meta-analysis demonstrated that a preoperative diet (LCD or VLCD) significantly reduced weight [SMD = − 0.68; 95% CI (− 0.93, − 0.42), *I*^2^ = 82%, *p* ≤ 0.01] and liver volume [SMD = − 2.03; 95% CI (− 4.00, − 0.06), *I*^2^ = 94%, *p* ≤ 0.01]. When assessed individually, a VLCD led to significant weight reduction [SMD = − 0.79; CI (− 1.24; − 0.34),* p* ≤ 0.01, *I*^2^ = 90%], as did an LCD [SMD = − 0.60; CI (− 0.90; − 0.29), *p* ≤ 0.01, *I*^2^ = 68%). Similarly, there was a significant reduction in liver volume following a VLCD [SMD = − 1.40; CI (− 2.77, − 0.03), *p* ≤ 0.01, *I*^2^ = 96%], and an LCD [SMD = − 2.66; CI (− 6.13, 0.81), *p* ≤ 0.01, *I*^2^ = 93%]. However, there was no significant difference between the two regimens.

**Conclusions:**

Preoperative restrictive calorie diets are effective in reducing weight and liver volume prior to laparoscopic surgery. Whilst a VLCD was better than an LCD at reducing both weight and liver volume, the difference was not significant.

**Graphical abstract:**

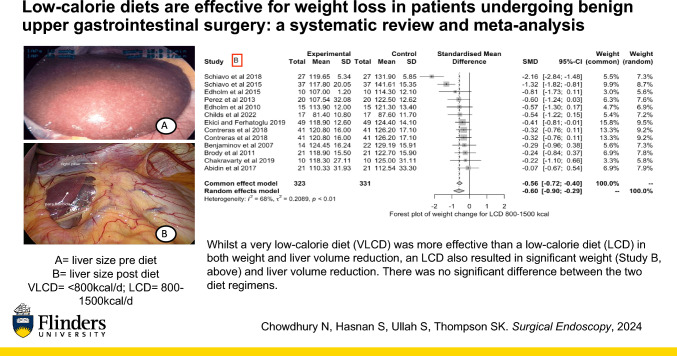

**Supplementary Information:**

The online version contains supplementary material available at 10.1007/s00464-024-11016-1.

Obesity is a major risk factor for many chronic diseases like cardiovascular disease, type 2 diabetes, and some cancers [1]. In 2015, 8.4% of the burden of disease in Australia was attributed to being overweight and/or morbidly obese [1]. Moreover, one in three Australians were found to be obese in 2017–2018 which is a 12% increase from 1995.

Laparoscopic surgery in obese patients poses challenges in visualization of structures in the surgical field and threatens damage to surrounding structures. It is also associated with adverse peri-operative outcomes such as anastomotic leak, poor healing, and longer operating times [2, 3]. As well, fatty liver disease is highly prevalent in patients who are obese which can add to the surgical challenges mentioned above [4].

Current literature recommends the use of preoperative very low-calorie diets to reduce weight and liver volume to improve operative access [2, 3, 5–27]. A variety of diets with different compositions are currently being used by surgeons for preoperative weight loss. These include very low-calorie diet (VLCD, < 800 kcal) [12, 17, 18, 21, 22], very low-calorie ketogenic diet (VLCKD, ~ 400 kcal) [23], ketogenic micronutrient-enriched diet (KMED, 1150–1500 kcal) [24], Mediterranean-protein-enriched diet (MPED, 1200 kcal) [28], low-energy diet (BCM, ~ 900 kcal) [26], low-carbohydrate diet (low carb, 1500 kcal) [9], and low-calorie diet (LCD, 800–1500 kcal) [8, 10, 11, 13–16, 19, 20, 25–27]. Diets with over 800 kcal per day seem to be better tolerated by patients prior to surgery but limited systematic reviews exist to compare efficacy of the various diets.

The aim of our study was to evaluate the efficacy of an LCD compared to a VLCD in reduction of weight and liver volume in patients prior to benign upper gastrointestinal surgery. Secondary aims included analyses of modality used for liver imaging, diet tolerance, and whether the use of a preoperative diet improved surgical outcomes.

## Methods

This review and meta-analysis fulfilled the proposed suggestions of the Cochrane Handbook for Systematic Reviews and Interventions [29] and was written using the PRISMA 2020 systematic review and meta-analysis guidelines [30] (Supplementary Fig. 1).

### Databases

A methodical search was conducted on five different databases (PubMed, OVIDSP [Medline, JBI, PsychInfo], CINAHL, Cochrane and Scopus) to recognize relevant primary studies. For the preliminary search, broad terms such as [very low-calorie diet], [low-calorie diet], [liver volume], [weight loss], and [pre-surgery] were used. Subsequently, the synonyms of the above terms were used to conduct the final search on 21 November, 2022 (Supplementary Table 1). Identified studies were entered into COVIDENCE for a second reviewer. In addition, the references of the collected articles were manually explored to identify other relevant studies.

### Criteria for selection

Inclusion criteria were the following: (1) primary articles written in English with full text available, (2) studies that prescribed a very low-calorie diet or low-calorie diet to achieve preoperative weight loss and liver volume reduction in patients planned for benign upper gastrointestinal surgery, (3) studies that included an objective measure of weight reduction (weight loss or body mass index (BMI) loss) and liver volume reduction (whole liver, left liver lobe, or visceral fat of liver) with an imaging modality (magnetic resonance imaging [MRI], computed tomography [CT], or ultrasound [US]), and (4) studies published between 1995 and 2022 with a sample size of at least 10 patients. Studies identified as reviews, non-peer-reviewed articles, conference abstracts, letters to editors, animal studies, using alternative methods of weight loss prior to surgery, including patients who underwent non-benign surgeries (i.e. cancer patients) were excluded. Following the systematic review, any studies missing weight reduction (in kg) and/or liver volume reduction (mL) raw values were excluded from the meta-analysis.

### Study quality assessment: individual study risk of bias and publication bias

Two independent reviewers applied the Cochrane risk of bias tool [31] for randomized controlled trials (RCTs) and an adapted version of the methodological quality checklist outlined by Downs and Black [32] for non-RCTs to evaluate individual study risk of bias. For the Cochrane risk of bias tool, studies were classified as low risk (≥ 4 low-risk domains), moderate risk (≥ 3 unclear risk domains), and high risk [(≥ 2 high-risk domains) (adapted from a similar systematic review [6])] based on their risk of bias. Following the modified Downs and Black checklist, a score between 20 and 22 was considered excellent, between 15 and 19 was considered good, between 11 and 14 was considered fair, and less than 11 was considered a poor-quality study (adapted from similar systematic review [6]). Any discrepancies between the two reviewers were subsequently discussed with the senior author and resolved. Publication bias was assessed with funnel plot asymmetry [33].

### Data extraction

Data extraction was completed by one author and subsequently reviewed by a second investigator. Extracted data were entered into a pre-determined global data entry table which contained the following headings: authors, location, publication year, type of study, sample size, breakdown of sample size by sex (if available), diet used, calories, duration of diet, baseline mean weight/BMI, change in mean weight/BMI after diet (in kg or kg/m^2^), baseline mean liver volume, change in mean liver volume (in mL), methods of liver imaging (liver, left liver lobe, visceral fat of liver), and modality used (MRI, CT, US). Moreover, any secondary outcomes included in the collected studies were also compiled, such as tolerance of diet and view of surgical field.

### Data synthesis and statistical analysis

Primary outcomes for this study included weight reduction and liver volume reduction from baseline to the patients’ scheduled benign upper gastrointestinal surgery. Secondary outcomes comprised the modality of liver imaging, surgical outcomes, and diet tolerance. Weight reduction and liver volume reduction were recorded in kg and mL, respectively. Studies that reported BMI change, baseline weight, and weight post diet were calculated by multiplying the baseline BMI and post-diet BMI with the height^2^ (if available). If studies reported median and confidence intervals, the median was considered equivocal to the mean and the IQR was divided by 1.35 to calculate the standard deviation.

The meta-analysis was undertaken with Review Manager 5.4.1 (RevMan). Standardized mean differences (SMD) with a 95% confidence interval (CI) were calculated and the statistical heterogeneity between studies was assessed using *I*^2^ statistics. Hedge’s *g* statistic was used to calculate the combined effect of preoperative diet on weight loss and liver volume reduction to account for the varied sample sizes amongst studies. To capture the impact of study heterogeneity, a random-effects model was applied for all studies and a *p* value of < 0.05 was deemed significant. A meta-regression analysis was performed to explore the impact of various factors on overall estimates. In addition, an influence analysis with a leave-one-out strategy was used to assess the influence of each study on the final combined outcome. The trim and fill method was used to address publication bias.

## Results

**Fig. 1:**
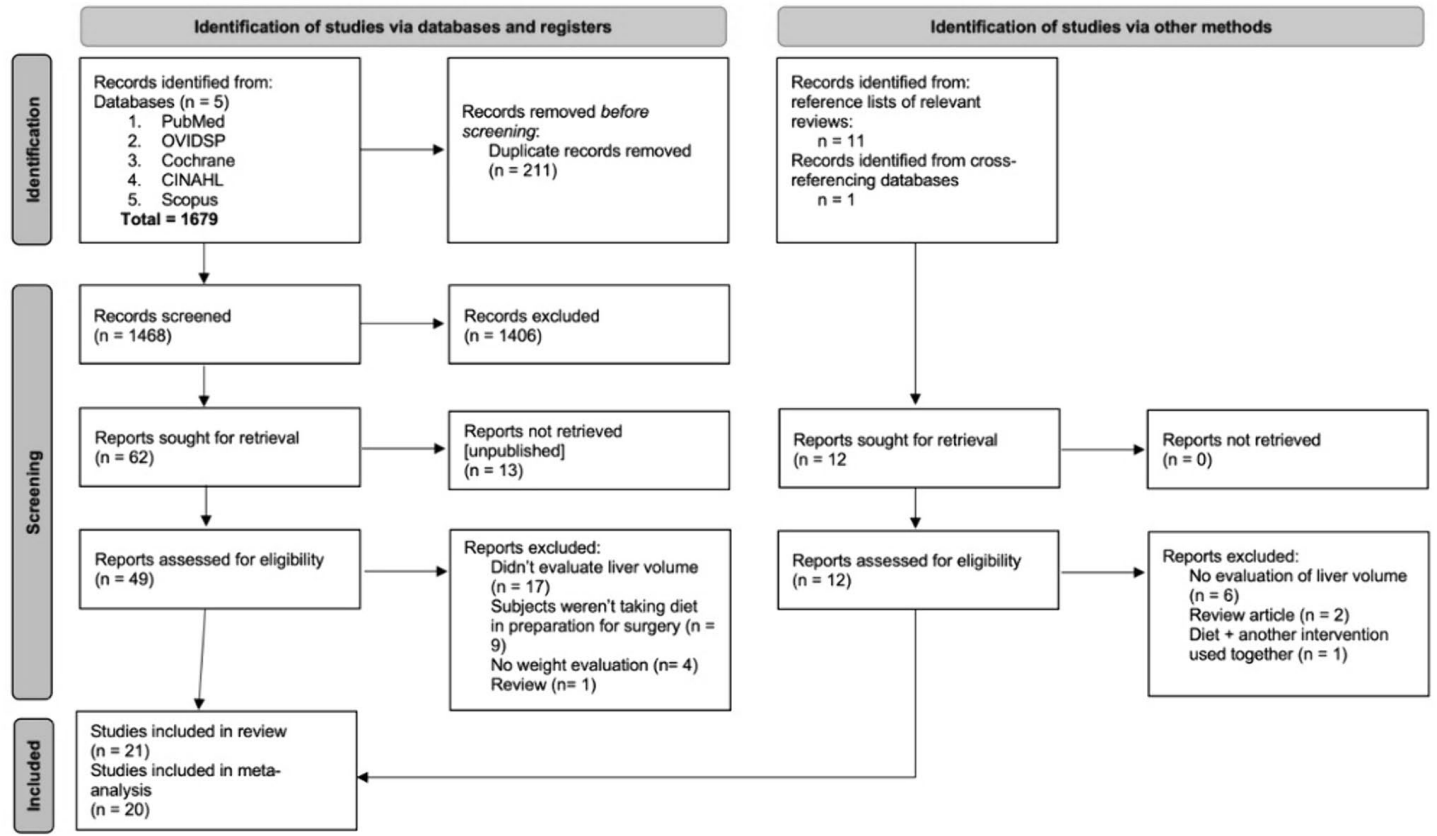
PRISMA flow diagram showing the study selection process

A total of 1679 studies were found using the search terms described in the Methods section. In addition, 12 studies were added from reference lists of relevant reviews. All studies were uploaded onto COVIDENCE (a web-based streamlined collaborative platform for systematic reviews) and screened by two independent reviewers. After 211 duplicates were removed, titles and abstracts of 1468 studies were screened for relevance; 1406 studies were excluded. Full-text review of 68 studies was conducted to finally include 21 studies for the systematic review and 20 studies for the meta-analysis (Fig. 1).

### Study characteristics and demographics

Of the 21 selected studies, 8 studies were single-subject research (i.e. the baseline measurements of each patient served as controls) [9, 15, 18, 20, 21, 23, 27, 28]. In addition, 8 studies were open-labelled, RCTs [8, 11, 14, 17, 19, 22, 25, 26], of which 3 trials researched the effects of two different diets on weight and liver volume reduction. Contreras et al. compared VLCD with LCD [19], Faria et al. compared normal consistency VLCD with a liquid VLCD [17], and Lange et al. compared low-energy diet (BCM) to Optifast® [26]. Moreover, 3 studies were prospective observational studies [10, 12] of which one study was a pilot study [24] (Table 1). Finally, 2 studies were retrospective reviews [13, 16]. A combined sample size of 814 patients was included in this systematic review: 544 females (67%) and 270 males (33%) (Table 1) with a mean age ranging from 24 to 54 years old. Studies originated from Australia, United States of America, Mexico, Spain, Netherlands, United Kingdom, Italy, Brazil, Sweden, Germany, Turkey, Israel, New Zealand, and Malaysia.

**Table 1:** Overview of included studies and patient demographics

Study no	Authors	Study type	Location, year	Sample size (n)	Gender F:M	Age (years) mean ± SD or median (IQR)
1	Lewis, CM et al. [21]	Single-subject study	Australia, 2006	18	17:1	50 (34–57)
2	Edholm et al. [15]	Single-subject study	Sweden, 2015	10	10:0	42.9 ± 8.9
3	Perez et al. [20]	Single-subject study	Mexico, 2013	20	17:3	34.5 ± 11.5
4	Contreras et al. [19]	RCT, open labelled	Spain, 2018	43	29:14	45.2 ± 10.5
5	Contreras et al. [19]	RCT, open labelled	Spain, 2018	41	34:7	45.5 ± 9.7
6	Bakker et al. [8]	RCT, open labelled	Netherlands, 2019	26	26:0	44 ± 18
7	Ministrini et al. [22]	RCT, open labelled	Italy, 2019	52	34:18	49 ± 12.5
8	Pilone et al. [23]	Single-subject research	Italy, 2018	119	75:44	43.6 ± 9.8
9	Edholm et al. [14]	RCT, open labelled	Sweden, 2011	15	15:0	42.2 ± 7.05
10	Colles et al. [12]	Prospective observational study	Australia, 2006	32	13:19	47.5 ± 8.3
11	Ekici and Ferhatoglu [16]	Retrospective cohort study	Turkey, 2019	49	32:17	37.4 ± 9.2
12	Brody et al. [10]	Prospective observational study	USA, 2011	21	17:4	47.6 ± 9.5
13	Collins et al. [13]	Retrospective review	USA, 2011	29	3:26	53 (34–53)
14	Benjaminov et al. [9]	Single-subject study	Israel, 2007	14	9:5	24–45
15	Schiavo et al. [24]	Prospective pilot study, single subject	Italy, 2018	27	17:10	41 ± 16.7
16	Fris [18]	Single-subject study	New Zealand, 2004	40	36:4	41 (25–74)
17	Abidin et al. [25]	RCT, open labelled	Malaysia, 2017	21	12:9	41
18	Faria et al. [17]	RCT, open labelled (liquid)	Brazil, 2015	57	45:12	37.14 ± 10.29
19	Faria et al. [17]	RCT, open labelled (normal consistency)	Brazil, 2015	47	40:7	36.43 ± 10.01
20	Chakravarty et al. [11]	RCT, open labelled	UK, 2019	10	10:0	43.5 (26–60)
21	Childs et al. [27]	Single-subject study	Australia, 2022	17	6:11	54 (21–74)
22	Schiavo et al. [28]	Prospective cohort study	Italy, 2015	37	0:37	46 ± 7.09
23	Lange et al. [26]	RCT, open labelled (BCM)	Germany, 2022	33	22:11	47.2 ± 11.5
24	Lange et al. [26]	RCT, open labelled (Optifast®)	Germany, 2022	36	25:11	46.3 ± 8.8
	Overall			814	544:270	Mean age range: 24–54 years

*RCT* randomized controlled trial

Of the 8 RCTs, two studies had a low risk of internal bias [11, 25] whilst the remainder had a moderate risk of bias [8, 14, 17, 19, 22, 26]. Three of the moderate-risk studies [14, 17, 22] included randomization bias, one study contained bias in ‘deviation from intended’ [26], and the remaining two studies [8, 19] contained detection bias (Supplementary Fig. 2). All of the RCTs were open label which increased their risk of bias for ‘allocation concealment’ and ‘random sequence generation’ categories (Suppl Fig. 2).

Of the non-RCT studies, one study was deemed excellent quality [12], eleven studies were rated as ‘good’ quality [9, 10, 15, 16, 20, 23, 24, 27, 28], and one study was rated as a ‘fair’ quality study [21]. Lewis et al. [21] demonstrated high risk of bias in ‘reporting’, ‘internal validity’, and ‘selection bias’ (Suppl Table 2). Overall, all non-RCT studies lacked blinding of participants and assessors except Fris et al. [18]. Finally, twelve of the thirteen studies did not calculate sufficient power to detect a clinically important effect, introducing a high risk of bias in ‘power’ (Supplementary Table 2).

**Fig. 2:**
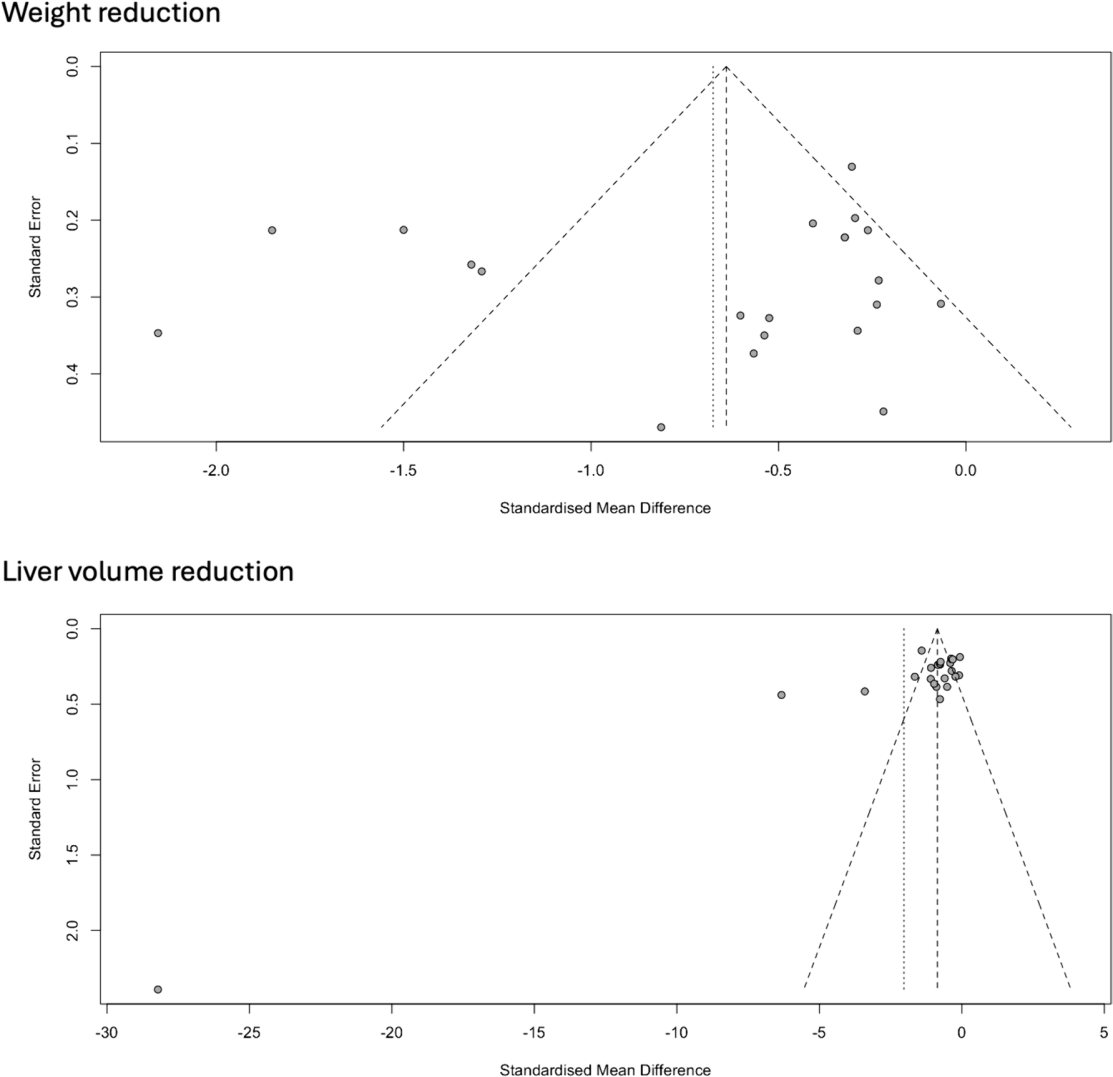
Funnel plots of meta-analysis for weight and liver volume reduction of included studies denoting publication bias

Asymmetry was evident in the meta-analysis funnel plots which denoted a publication bias in the spectrum of studies published in this area (Fig. 2). Studies that demonstrated lower effects (low standard mean difference) of preoperative diet on weight reduction were more likely to be published compared to studies that showed a higher effect. However, the meta-analysis funnel plot for preoperative diet effect on liver volume reduction showed a more symmetrical distribution of studies which demonstrated a comparatively lower publication bias. The trim and fill method failed to adjust for publication bias in our meta-analysis as the adjusted effect size remained similar to the original estimate.

### Preoperative diet

A variety of preoperative diets with a multitude of constituents and supplements were used by the studies included in this systematic review (Table 2). Many of the studies (twelve out of twenty-one studies) used an LCD formulation prior to surgery [8, 10, 11, 13–16, 19, 20, 25–27]. Another six studies used a very low-calorie diet [12, 17–19, 21, 22], and the final five studies used novel diet regimens, i.e. VLCKD [23], KMED [24], MPED [28], BCM [26], and low carb [9]. One study compared the effects of VLCD with LCD [19] and another compared BCM to Optifast® [26]. The median duration for the diets was 4 weeks (range: 10–84 days) (Table 2). Diets varied in their calories, consistency, and composition. Sixteen studies prescribed a liquid diet [8, 10–15, 17–23, 26, 27], five studies prescribed a normal consistency diet [9, 17, 24, 25, 28], and one study did not include any description on the consistency of their diet [16]. Of the liquid consistency diets, ten diets were solely liquid, and eight diets were a combination of liquid and normal consistency, with one study comparing the effects of liquid and normal consistency diet on weight loss [17].

**Table 2:** Composition of preoperative diets

Authors	Diet composition					
	Intervention	Control	Calories (Kcal/d)	Duration (weeks)	Consistency of diet	Meal replacement or supplement
Lewis et al. [21] VLCD	Optifast® (Novartis, consumer health Australasia Pty, Ltd) 17 g protein, 2.3 g fat, 15 g CHO/sachet	Compared to baseline	400–800	6	Liquid + food	Replacement + low-calorie food
Edholm et al. [15] LCD	Modifast® (Impolin AB, Stockholm, Sweden) 52% CHO, 25% protein, 21% fat	Compared to baseline	800–1100	4	Liquid	Replacement
Perez et al. [20] LCD	20% CHO, 34% protein, 46% mono- and polyunsaturated fats + 9.5 g of fibre, 478mg of sodium, 1.664 mg potassium and 838mg phosphate	Compared to baseline	800	6	Liquid + food	Replacement + low-calorie food (68 g protein, 40 g fats, 40 g CHO)
Contreras et al. [19] VLCD	Optifast® (Nestlé health science, 2011) 46.8% CHO, 36.4% protein, 9.3% fat and 7.4% fibre	Compared to LCD and baseline	< 800	3	Liquid	Replacement + broth + non-calorie beverages
Contreras et al. [19] LCD	As above	Compared to VLCD and baseline	1200	3	Liquid + food	Supplement + food provided
Bakker et al. [8] LCD	Omega-3 fatty acid capsules (1000mg, Teva BV, the Netherlands) 840 mg ethyl esters of EPA (460 mg) and DHA (380 mg)	Modifast® shakes 52% CHO, 25% protein, 21% fat	2000 for fatty acid group	4 for fatty acid group; 2 for LCD	Liquid + food	Supplement + healthy diet
Ministrini et al. [22] VLCD	Nepicomplex for protein + Solus multinutrient + xaliform < 50 g CHO, 1.4 g/kg of ideal weight protein intake, < 30 g fat	Sham diet (no mention of details)	< 800	3.6	Liquid + food	Replacement + low-calorie food
Pilone et al. [23] VLCKD	KetoStationKit (Nutri&biotech srl, Castelnuovo Rangone Italy) 0.3 g of carbohydrate, 8.4 g protein, 0.4 g of fat/scoop	Compared to baseline	400	1.43	Liquid + food	Replacement + food
Edholm et al. [14] LCD	Modifast® (Impolin AB, Stockholm, Sweden) 52% CHO, 25% protein, 21% fat	Not described	800–1100	4	Liquid	Replacement
Colles et al. [12] VLCD	Optifast® (Novartis, consumer health Australasia Pty, Ltd) 52 g protein, 45 g CHO, 7 g fat + 250 g low-starch vegetables	Compared to baseline	456- 680	12	Liquid + food	Supplement + low starch vegetables
Ekici and Ferhatoglu [16] LCD	Individualized diet plans → nil description	Compared to baseline	1000	4	Nil description	Nil description
Brody et al. [10] LCD	Nuvista (Nutricia Liverpool, Trading division of SHS International Ltd., London, UK) 15 g protein, 17 g CHO (5 g of fibre), 2.5 g fat + vitamin D, calcium, iron per serving	Compared to baseline	1200–1500	4	Liquid	Supplement
Collins et al. [13] LCD	Optifast® (Nestlé health science, 2011) 46.8% CHO, 36.4% protein, 9.3% fat and 7.4% fibre	Compared to baseline	800	9	Liquid	Replacement
Benjaminov et al. [9] Low-carbohydrate diet	Low-carbohydrate diet 110 g ± 36 proteins, 54 g ± 22 CHO, 94 g ± 22 fat	Compared to baseline	1520	4	Normal consistency	Replacement
Schiavo et al. [24] KMED	4% CHO, 71% fats, 25% protein + Ketocompleat (MVMedical solutions, Serraville, Repubblia San Mariono)	Compared to baseline	1150–1250	4	Normal consistency	Replacement + Supplement
Fris [18] VLCD	Optifast® (Novartis, consumer health Australasia Pty, Ltd) 52 g protein, 45 g CHO, 7 g fat	Compared to baseline	456	2	Liquid	Replacement
Abidin et al. [25] LCD	Omega-3 polyunsaturated fatty acids (PUFAs) Nil description	VLCD Nil description	800	4	Normal consistency	Replacement
Faria et al. [17] VLCD	78 ± 3 g Protein, 20 ± 1 g fats, 67 ± 2 g CHO + whey supplement (20 g protein)	Compared to normal consistency VLCD (below)	760 ± 26	2	Liquid	Replacement + Supplement
Faria et al. [17] VLCD	73 ± 4 g protein, 19 ± 1 g fats, 71 ± 4 g CHO + whey supplement (10 g protein)	Compared to liquid VLCD (above)	754 ± 23	2	Normal consistency	Replacement + Supplement
Chakravarty et al. [11] LCD	Cambridge Milk diet [34] 82 g CHO, 61 g Protein, 30 g fats + multivitamin and mineral supplement	Compared to normal diet (no description)	800	4	Liquid	Replacement
Childs et al. [27] LCD	Optifast® VLCD, Optislim® VLCD, or Proslim Rapid VLCD	Compared to baseline	1042.35	3.9	Liquid + food	Replacement + low-calorie food and beverages
Schiavo et al. [28] MPED	CHO 141 g, fat 35 g, protein 80 g, fibre 30 g, 2 L of low-sodium water	Compared to baseline	1200	8	Normal consistency	Replacement
Lange et al. [26] BCM	BCM Diät™ (PreCon GmbH, Darmstadt) Protein 75 g, fat 28 g, CHO 80 g	Compared to Optifast® (below)	913	2	Liquid	Replacement
Lange et al. [26] Optifast®	Optifast® (Nestle HealthCare Nutrition GmbH, München) Protein 58 g, fat 19 g, CHO 111 g	Compared to BCM (above)	841	2	Liquid	Replacement

*LCD* low-calorie diet, *VLCD* very low-calorie die, *CHO* carbohydrate, *EPA* eicosapentaenoic acid, *DHA* docosahexaenoic acid, *VLCKD*  very low-calorie ketogenic diet, *KMED* ketogenic micronutrient-enriched diet, *MPED* Mediterranean-protein-enriched diet, *BCM* low-energy diet

### Reduction in weight and liver volume

All studies reported a reduction in weight after consumption of the preoperative diet prescribed (Table 3). Mean baseline weight was 121.5 kg ± 16 kg and mean post-diet weight was 114 kg ± 16 kg. The mean %weight loss post diet was 6.42%. The mean baseline liver volume was 1,644 mL ± 403 mL with a post-diet mean liver volume of 1,328 mL ± 305 mL. The resulting average %loss in liver volume equated to 16.7% (Table 4).

**Table 3:** Post-diet weight loss

Study authors	Mean baseline weight (kg)	Post-diet mean weight (kg)	% Change
Lewis et al. [21]	119.7	110.6	7.6
Edholm et al. [15]	114.3	107	6.47
Perez et al. [20]	122.5	107.54	12.27
Contreras et al. [19]	131.2	123.6	5.81
Contreras et al. [19]	126.2	120.8	4.19
Bakker et al. [8]	117	111.63	4.59
Ministrini et al. [22]	122	115	5.73
Pilone et al. [23]	117.4	109.9	6.30
Edholm et al. [14]	121.3	113.9	6.10
Colles et al. [12]	139.8	125	10.60
Ekici and Ferhatoglu [16]	124.4	118.9	4.42
Brody et al. [10]	122.7	118.9	3.10
Collins et al. [13]^a^	56 kg/m^2^	49 kg/m^2^	12.30
Benjaminov et al. [9]	129.19	124.45	3.67
Schiavo et al. [24]	131.9	119.65	9.29
Fris [18]	125.275	120.07	4.10
Abidin et al. [25]	112.54	110.33	1.96
Faria et al. [17]	114.46	110.7	3.49
Faria et al. [17]	107.41	104.61	2.61
Chakravarty et al. [11]	125	118.3	5.36
Childs et al. [27]	87.6	81.4	7.08
Schiavo et al. [28]	141.61	117.8	16.81
Lange et al. [26]^a^	47.5 kg/m^2^	45.69 kg/m^2^	3.81
Mean overall	121.5	114	6.42

^a^Only BMI reported

*BMI* body mass index

**Table 4:** Post-diet liver volume reduction

Study authors	Mean baseline liver volume (mL)	Post-diet mean liver volume (mL)	% change	% change/week
Lewis et al. [21]	2199	1934	12.05	2.01
Edholm et al. [15]	2100	1700	18.0	4.50
Perez J.G. et al. [20]	2294.78	1829.51	20.28	3.38
Contreras et al. [19]	2653	2208	15.60	5.20
Contreras et al. [19]	2600	2268	12.30	4.10
Bakker et al. [8]	2137	1866	12.68	6.34
Ministrini et al. [22]	264	235	10.98	3.05
Pilone et al. [23]	263.5	209	20.60	14.41
Edholm et al. [14]	2170	1890	12.90	3.23
Colles et al. [12]	2800	2300	18.70	1.56
Ekici and Ferhatoglu [16]	201.64	158.76	11.27	2.82
Brody et al. [10]	562.5	299.9	43.40	10.85
Collins et al. [13]	3385.5	2711.4	18.0	2.00
Benjaminov et al. [9]	2718	2495	8.20	2.05
Schiavo et al. [24]	627	503	19.78	4.95
Fris [18]	Area: 88.16	73.44	5.10	2.55
Abidin et al. [25]	1647.04	1609.94	2.25	0.006
Faria et al. [17]	82.5	65.5	20.60	10.30
Faria et al. [17]	71.3	71.1	0.28	0.14
Chakravarty et al. [11]	Nil reported	Nil reported	23.0	5.75
Childs et al. [27]	1776.65	1459.11	17.87	4.58
Schiavo et al. [28]	294.88	156.31	46.99	5.87
Lange et al. [26]	2687	2230	17.01	8.50
Lange et al. [26]	2634	2292	12.98	6.49
Mean overall	1644	1328	16.7	4.78

### Imaging modalities

Selected studies used various modalities to evaluate the decrease in liver volume post low-calorie diet. Most studies used ultrasound (43%), 22% used CT, and 35% used MRI (Table 5). Although most studies evaluated whole liver volume [8, 9, 12–15, 19–21, 25–27], some studies evaluated liver volume using proxy measures such as left liver lobe volume [10, 11, 16, 18, 24], visceral and/or intrahepatic fat [17, 22], right and left liver lobe volume [28], and caudate lobe volume [23].

**Table 5:** Imaging modalities used

Study authors	US	CT	MRI	Method used
Lewis et al			X	Liver volume + liver fat
Edholm et al			X	Liver volume + liver fat
Perez et al		X		Liver volume
Contreras et al		X		Liver volume
Bakker et al			X	Liver volume + left liver lobe + liver fat
Ministrini et al	X			Liver visceral fat
Pilone et al	X			Right liver lobe + caudate lobe
Edholm et al			X	Liver + intrahepatic fat
Colles et al		X	X	Liver volume
Ekici and Ferhatoglu	X			Left liver lobe
Brody et al	X			Left lateral segment of the liver
Collins et al		X		Liver volume + visceral adipose tissue
Benjaminov et al		X		Liver density + liver volume
Schiavo et al.	X			Left liver lobe volume
Fris	X			Left lobe of liver
Abidin et al.			X	Liver volume
Faria et al.	X			Visceral fat of liver
Chakravarty et al.	X			Left lobe
Childs et al.	X		X	Liver volume
Schiavo et al.	X			Liver lobes individually
Lange et al.			X	Liver volume
Overall percentage of total	43	22	35	N/A

*US* ultrasound, *CT* computed tomography scan, *MRI* magnetic resonance imaging

### Diet tolerance and compliance

Diet tolerance was included in fourteen studies [9–11, 14, 18, 21, 27]. If significant intolerance issues occurred, patients were able to withdraw from their respective programs and/or were counselled through the issues [8, 13, 21]. Common adverse effects reported by participants included musculoskeletal pain, constipation, headaches, urge to chew, nausea, diarrhoea, asthenia, and light headedness [8, 10, 12, 15, 19, 20, 23, 24, 28] (Supplementary Table 3). Objective measurement of compliance to diet was performed by assessment of ketonuria at regular monitoring visits (42% of studies) [12, 15–17, 20, 22–24, 28] and counting empty sachet packets of VLCD and LCD [19]. Subjective measures of diet compliance included patient interviews [8], food diary [25, 26], 3-day food records and 72h recalls [17, 24, 28], and history at check-up appointments [13].

### Biochemical panels and surgical complications

All studies, except one [27], evaluated baseline and post-diet biochemical parameters. Most studies demonstrated a reduction in systolic and diastolic blood pressures post low-calorie diet. In addition, marked reductions were observed in low-density lipoprotein, glucose, and insulin. Some studies reported on the technical attributes of surgery and surgical outcomes post diet (i.e. ease of access, liver retraction, peri-operative complications, conversion to open procedure, operative time, and hospital stay) [9–14, 16, 17, 21] (Supplementary Table 3). Post diet, there was improved access to the gastro-esophageal junction [9, 13, 21], a smaller liver [9, 21], little to no major post-operative complications [10, 12–14, 19], less conversions to open procedures [12], and reduction in overall operative time [13, 14, 17]. However, only one study demonstrated a reduction in hospital stay post diet [16], whilst four did not [11, 12, 14, 19].

### Meta-analysis

**Fig. 3:**
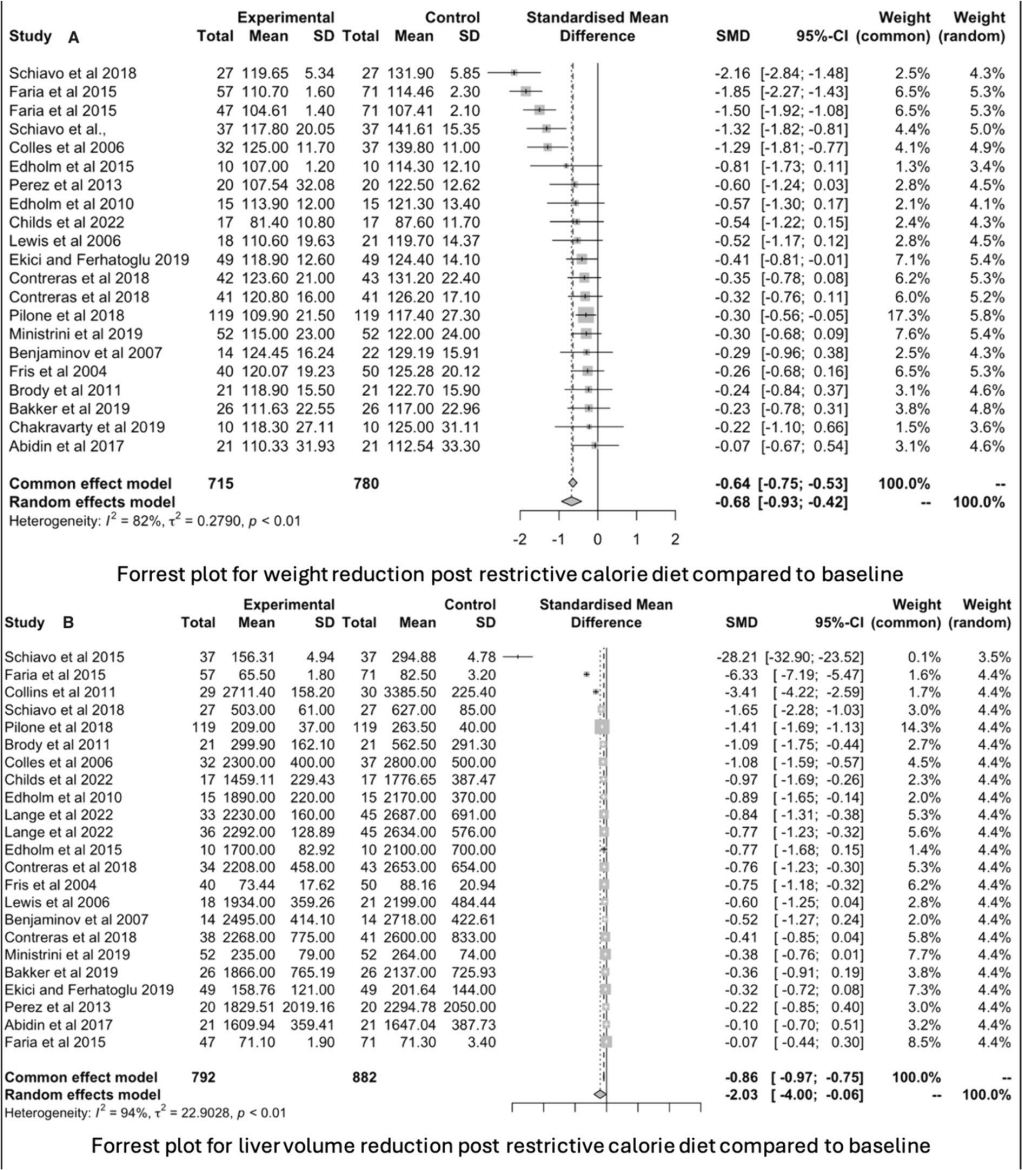
Forest plots of meta-analysis of weight (Study A) and liver volume (Study B) reduction from baseline to post-diet follow-up. A preoperative diet significantly reduced weight (Study A) and liver volume (Study B)

The main outcome for the meta-analysis was a reduction in weight and liver volume post diet compared to baseline measurements (pre-diet or controls). Compared to baseline, a preoperative diet (VLCD, VLCKD, LCD, KMED, low carb) led to significant weight reduction prior to benign upper gastrointestinal surgery [SMD = − 0.68; confidence interval (− 0.93, − 0.42), *p* ≤ 0.01; *I*^2^ = 82%] (Fig. 3, Study A). There was a significant reduction in liver volume after consumption of a preoperative diet compared to baseline [SMD = − 2.03; 95% CI (− 4.00, − 0.06), *p* ≤ 0.01, *I*^2^ = 94%] (Fig. 3, Study B).

**Fig. 4:**
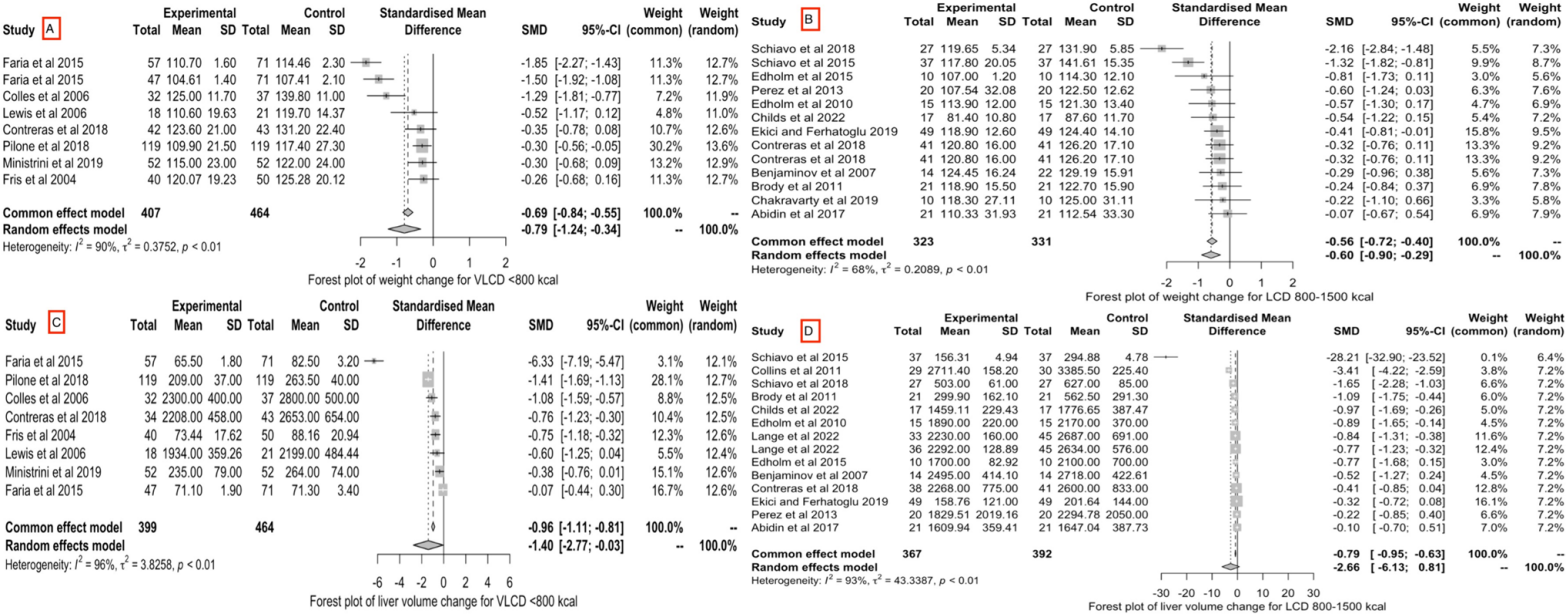
Forest plots of meta-analysis comparing weight reduction from baseline to post very low-calorie diet (VLCD) (Study A) and low-calorie diet (LCD) (Study B), and liver volume reduction from baseline to post VLCD (Study C) and LCD (Study D). Both VLCDs and LCDs showed a significant reduction in weight and liver volume. Whilst VLCDs showed greater efficacy in reducing weight and liver volume compared to LCDs, this difference was not statistically significant

When assessed individually, a VLCD led to significant weight reduction [SMD = − 0.79; CI (− 1.24; − 0.34), *p* ≤ 0.01, *I*^2^ = 90%] (Fig. 4, Study A), as did an LCD [SMD = − 0.60; CI (− 0.90; − 0.29), *p* ≤ 0.01, *I*^2^ = 68%] (Fig. 4, Study B). Similarly, there was a significant reduction in liver volume following a VLCD [SMD = − 1.40; CI (− 2.77, − 0.03), *p* ≤ 0.01, *I*^2^ = 96%] (Fig. 4, Study C), and an LCD [SMD = − 2.66; CI (− 6.13, 0.81), *p* ≤ 0.01, *I*^2^ = 93%] (Fig. 4, Study D). That said, whilst VLCDs showed greater efficacy in reducing weight and liver volume compared to LCDs, this difference was not statistically significant (Fig. 4).

A multivariate meta-regression analysis was performed to explore the impact of diet composition, diet duration, and research design on the final results. No significant difference was found in heterogeneity between the meta-regression analysis and the standard random-effects meta-analysis model (*I*^2^ = 81.6% for weight, *I*^2^ = 99.7% for liver volume), suggesting that these factors did not significantly account for the differences in true effect sizes. Similarly, an influence analysis was done to evaluate the impact of each study on the overall combined outcome. The SMDs for weight and liver volume reduction remained the same regardless of which study was omitted from the analysis. The combined effects (− 0.67 for weight reduction and − 2.03 for liver volume reduction) remained consistent when using the leave-one-out strategy.

## Discussion

Whilst our meta-analysis showed that a VLCD was more effective in both weight and liver volume reduction prior to benign upper gastrointestinal surgery, an LCD also resulted in significant weight and liver volume reduction, and there was no significant difference between the two diet regimens. This is an important finding as LCDs are often better tolerated than VLCDs, increasing patient compliance.

Amongst the five different diet regimens seen in our systematic review, a VLCKD demonstrated the greatest efficacy in reducing weight per week of the diet used [23]. This is not surprising given VLCKDs portend less than 400 kcal per day [3]. The highest weight loss overall was not associated with the longest duration of the preoperative diet, nor the lowest calories consumed [13]. Collins et al. demonstrated excellent reduction in baseline weight with an LCD set at 800 kcal/day, just over the threshold of a VLCD. That said, patients stayed on this diet for 9 weeks, longer than many of the other studies set at 4 weeks or less. Although counterintuitive, the findings of this study are supported by previous primary studies [18, 21] and reviews [2, 5] where a minimum period of two weeks for a preoperative diet was sufficient to achieve significant weight loss in patients.

Other findings deserve mention. First, restricted calorie diets had excellent tolerance amongst patients with only 17 out of 814 patients declining to participate due to diet intolerance (2.1%) (Table 1, Figs. 3, and 4). This is supported by recent reviews investigating the effects of preoperative diets on weight loss prior to bariatric surgery [3, 5]. Second, a significant decrease in liver function across most studies supports the notion that restrictive calorie diets reverse liver steatosis [17, 22, 23, 26, 38]. Even though preoperative weight loss may provide an intra-operative surgical benefit, only five of 21 studies discussed better access to the hiatus, reduced surgical time, and easier liver retraction [9, 13, 14, 17, 21].

In terms of liver volume reduction, the greatest reductions (43% and 47%) were seen in studies that used a liquid LCD for 4 weeks [10] and an MPED for 8 weeks [28], respectively. This again was neither the longest duration nor the lowest number of calories consumed as the study used a nutritional supplement (Nuvista) rather than a meal replacement. These studies demonstrated liver volume loss of more than double that seen in other studies. It is possible that the authors’ measurement of liver volume with ultrasound was inaccurate as they used left liver lobe measurement only. This may not represent whole liver volume. A similar study showed only a 20% liver volume reduction [24].

Two studies reported liver volume reduction occurred during the first two weeks [15] and up to 4 weeks [20]. Surprisingly, the second study demonstrated increased liver volumes from weeks 4 to 6 even though the diet regimen did not change [20]; this could have been due to diet non-compliance. One study reported that 90% of their patients achieved maximal liver volume loss within 3 weeks of an LCD [27].

Many different imaging techniques were used in our systematic review ranging from MRI, CT, and ultrasound. Although no obvious trends emerged, MRI seemed the most accurate for predicting overall liver volume [8, 12, 14, 15, 21, 25–27]. This finding was supported by a recent review completed by van Wissen et al. [2]. CT is also very good at gauging liver volume; however, it has the drawbacks of expense and radiation exposure [35]. Finally, ultrasound has been shown to accurately measure degree of liver steatosis rather than volume [2, 35]. Ultrasound measurements of various segments of liver are operator dependent, and most studies do not use a validated method of calculating liver volume [10, 11, 16–18, 22–24, 28]. A recent study by Childs et al. has shown that ultrasound is just as accurate in the measurement of liver volume compared to MRI [27], using a validated technique and equation [36, 37].

There was great heterogeneity (weight reduction *I*^2^ = 82%, liver volume reduction *I*^2^ = 94%) in the meta-analysis amongst the studies which can be attributed to differences in baseline weight, number of calories consumed, duration of diet, consistency of diet, and whether the preoperative diet was administered as a supplement or replacement. Moreover, the imaging methods used also differed between studies.

Limitations of this systematic review include the heterogeneity of weight loss diets, unconventional presentation of data, lack of raw values, and lack of operator findings. For these reasons, the risk of interpretation bias was increased. Notable strengths of this study are use of PRISMA guidelines to conduct the review, use of PICO [patient, intervention, comparison, and outcome] formula to outline primary aims, use of two independent reviewers for data screening/collection/study appraisals, and the creation of standardized data collection tables to reduce individual reviewer bias.

## Conclusion

Our systematic review and meta-analysis showed that restrictive calorie diets, either VLCD or LCD, are effective in weight and liver volume reduction prior to benign upper gastrointestinal surgery. Whilst VLCDs are more effective in weight and liver volume reduction, there was no significant difference when compared to LCDs. Patient compliance and satisfaction may be improved with an LCD compared to a VLCD but, overall, we found excellent tolerance amongst patients.

### Supplementary Information

Below is the link to the electronic supplementary material.

Supplementary file1 (DOCX 143 KB)
