# Supplementary material for: Low-calorie diets are effective for weight loss in patients undergoing benign upper gastrointestinal surgery: a systematic review and meta-analysis
Source: Surg Endosc. 2024 Jul 8;38(8):4171–85. doi: 10.1007/s00464-024-11016-1 (PMC11289242; doi:10.1007/s00464-024-11016-1)
Supplement: Supplementary file 1 — Supplementary file1 (DOCX 143 KB) [file 464_2024_11016_MOESM1_ESM.docx]

**SUPPLEMENTARY FIGURES AND TABLES**

Supplementary Table 1: Overview of the search strategy.

| **Data Bases** | **Search Strategy** | **Results** |
| --- | --- | --- |
| PubMed | “VLCD” [Mesh] or “Optifast” [tiab] OR “VLCKD” [tiab] OR “Ketosis” [tiab] OR “Hypocaloric” [tiab] OR “Very Low-calorie Diet” [tiab] AND “Liver” [Mesh] or “Parenchyma” [tiab] AND “Weight Loss” [Mesh] OR “Change” [tiab] OR “Reduction” [tiab] OR “Volume change” [tiab] AND “pre-surgery” [Mesh] OR “Pre-OP” [tiab] OR “Prior to Surgery” [tiab] OR “Before Surgery” [tiab]. | 132 articles |
| OVIDSP (Medline, JBI, PsychInfo) | (Very low-calorie diet or VLCD or VLCKD or ketosis or energy restricted or hypocaloric). ab. and (Liver or parenchyma or volume).tx. and ((weight adj3 loss) or reduction or change). ab. and (surgery or pre-op or before surgery or prior to surgery or pre-surgery). ab. | 491 articles |
| Cochrane | “Very low-calorie diet” OR "VLCD" OR "VLCKD" OR "ketosis" OR "low carb*" OR "energy restricted" OR "hypocaloric" AND "liver size" OR "liver volume*" OR parenchyma AND "weight loss" OR "weight change" OR "weight reduction" AND "pre-surgery" OR "prior to surgery" OR "before surgery*" OR "preoperative" OR "pre-op*" | 650 articles |
| CINAHL | very low-calorie diet OR low-calorie diet OR VLCD or VLCKD OR ketosis OR low carb* OR hypocaloric OR energy restricted AND weight loss OR weight change OR weight reduction AND liver size OR liver volume OR parenchyma AND pre-surgery OR preoperative OR "prior to surgery" OR "before surgery" OR pre-op | 232 articles |
| Scopus | (ALL (very  AND low  AND calorie  AND diet  OR  low  AND calorie  AND diet  OR  vlcd  OR  vlckd  OR  ketosis  OR  low  AND carb*  OR  hypocaloric  OR  energy  AND restricted )  AND  ALL ( weight  AND loss  OR  weight  AND change  OR  weight  AND reduction )  AND  ALL ( liver  AND size  OR  liver  AND volume  OR  parenchyma )  AND  ALL ( pre-surgery  OR  preoperative  OR  "prior to surgery"  OR  "before surgery"  OR  pre-op ) ) | 174 articles |
| Articles from reference lists of relevant studies | Not applicable | 12 articles, 11 from reference list and 1 from cross referencing databases |


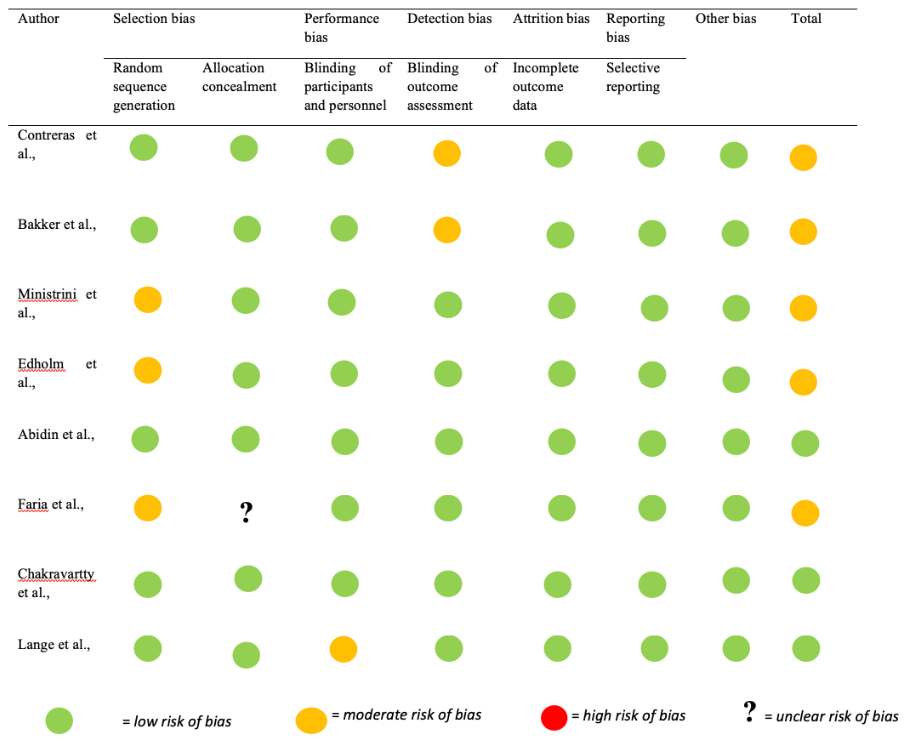


Supplementary Figure 1: Cochrane risk of bias tools to evaluate the methodologies of RCTs included in this study.

Supplementary Table 2: Downs and Black modified risk of bias tool for non-RCTs.

| **Downs and Black (modified): Reporting** | **Lewis, CM et al. 2006** | **Edholm, D et al. 2015** | **Perez J.G. et al. 2013** | **Pilone et al. 2018** | **Colles et al. 2006** | **Ekici & Ferhatoglu, 2020** | **Brody et al., 2011** | **Collins et al. 2006** | **Benjaminov et al. 2007** | **Schiavo et al. 2018** | **Fris et al. 2004** | **Childs et al. 2022** | **Schiavo et al. 2015** |
| --- | --- | --- | --- | --- | --- | --- | --- | --- | --- | --- | --- | --- | --- |
| Is the hypothesis/aim/objective of the study clearly described? | 1 | 1 | 1 | 1 | 1 | 1 | 1 | 1 | 1 | 1 | 0 | 1 | 1 |
| Are the main outcomes to be measured clearly described in the Introduction or Methods section? | 1 | 1 | 1 | 1 | 1 | 1 | 1 | 1 | 1 | 1 | 0 | 1 | 1 |
| Are the characteristics of the patients included in the study clearly described? | 1 | 1 | 0 | 1 | 1 | 1 | 1 | 1 | 0 | 1 | 1 | 1 | 1 |
| Are the interventions of interest clearly described? | 1 | 1 | 1 | 1 | 1 | 1 | 1 | 1 | 1 | 1 | 1 | 1 | 1 |
| Are the main findings of the study clearly described? | 1 | 1 | 1 | 1 | 1 | 1 | 1 | 1 | 1 | 1 | 1 | 1 | 1 |
| Does the study provide estimates of the random variability in the data for the main outcomes? | 1 | 1 | 1 | 1 | 1 | 1 | 1 | 1 | 1 | 1 | 1 | 1 | 1 |
| Have all important adverse events that may be a consequence of the intervention been reported? | 0 | 1 | 1 | 1 | 1 | 0 | 0 | 0 | 0 | 1 | 0 | 0 | 1 |
| Have the characteristics of patients lost to follow-up been described? | 1 | 1 | 1 | 1 | 1 | 1 | 1 | 0 | 1 | 1 | 1 | 1 | 1 |
| Have actual probability values been reported (e.g. 0.035 rather than <0.05) for the main outcomes except where the probability value is less than 0.001? | 1 | 1 | 1 | 1 | 1 | 1 | 1 | 1 | 1 | 1 | 1 | 1 | 1 |
| **External validity** |  |  |  |  |  |  |  |  |  |  |  |  |  |
| Were the subjects asked to participate in the study representative of the entire population from which they were recruited? | 1 | 1 | 1 | 1 | 1 | 1 | 1 | 1 | 1 | 1 | 1 | 1 | 0 |
| Were those subjects who were prepared to participate representative of the entire population from which they were recruited? | 0 | 0 | 0 | 1 | 1 | 1 | 1 | 0 | 1 | 1 | 0 | 1 | 0 |
| Were the staff, places, and facilities where the patients were treated, representative of the treatment the majority of patients receive? | 1 | 1 | 1 | 1 | 1 | 1 | 1 | 0 | 1 | 1 | 1 | 1 | 1 |
| **Internal validity - bias** |  |  |  |  |  |  |  |  |  |  |  |  |  |
| Was an attempt made to blind study subjects to the intervention they have received? | 0 | 0 | 0 | 0 | 0 | 0 | 0 | 0 | 0 | 0 | 0 | 0 | 0 |
| Was an attempt made to blind those measuring the main outcomes of the intervention? | 0 | 0 | 0 | 0 | 0 | 0 | 0 | 0 | 0 | 0 | 1 | 0 | 0 |
| If any of the results of the study were based on “data dredging”, was this made clear? | 1 | 1 | 1 | 1 | 1 | 1 | 1 | 1 | 1 | 1 | 1 | 1 | 1 |
| In trials and cohort studies, do the analyses adjust for different lengths of follow-up of patients, or in case-control studies, is the time period between the intervention and outcome the same for cases and controls? | 1 | 1 | 1 | 1 | 1 | 1 | 1 | 1 | 1 | 1 | 1 | 1 | 1 |
| Were the statistical tests used to assess the main outcomes appropriate? | 1 | 1 | 1 | 1 | 1 | 1 | 1 | 1 | 1 | 1 | 1 | 1 | 1 |
| Was compliance with the intervention/s reliable? | 1 | 0 | 1 | 0 | 1 | 1 | 1 | 0 | 1 | 1 | 1 | 1 | 1 |
| Were the main outcome measures used accurate (valid and reliable)? | 1 | 1 | 1 | 1 | 1 | 1 | 1 | 1 | 1 | 1 | 1 | 1 | 1 |
| **Internal validity - confounding (selection bias)** |  |  |  |  |  |  |  |  |  |  |  |  |  |
| Were losses of patients to follow-up taken into account? | 0 | 1 | 1 | 1 | 1 | 1 | 1 | 1 | 1 | 1 | 1 | 1 | 1 |
| Variety in patients? Confounders? Age, weight etc | 1 | 1 | 1 | 1 | 1 | 1 | 1 | 1 | 1 | 1 | 1 | 1 | 1 |
| **Power** |  |  |  |  |  |  |  |  |  |  |  |  |  |
| Did the study have sufficient power to detect a clinically important effect where the probability value for a difference being due to chance is less than 5%? | 0 | 0 | 0 | 0 | 0 | 0 | 1 | 0 | 0 | 0 | 0 | 0 | 0 |
| Overall | 16 | 17 | 17 | 18 | 19 | 18 | 19 | 14 | 17 | 19 | 16 | 18 | 17 |

Supplementary Table 3: Secondary outcomes and measure of diet compliance.

| **Study Authors** | **Secondary outcomes: adverse outcomes to diet or surgical outcomes** | **Diet compliance measures** |
| --- | --- | --- |
| Lewis, CM et al., | operative access (subjectively evaluated), access was thought to be easy and good post diet, easy retraction of soft liver | nil mention of evaluation, compliance issues subjective discussed with patients |
| Edholm, D et al., | SBP decrease, biochemical parameters decrease, fasting glucose, ALT unchanged, insulin level decrease, Cr increase, tolerance of diet + adverse effects of diet like hunger, urge to chew, MSK pain was measured but no surgical outcomes measured | ketonuria, presence of continuous weight loss |
| Perez J.G. et al., | anthropometric data, HR, BP, excess weight loss (lower post diet) was all measured. Tolerance of diet and acceptability of diet was also measured (taste, absence of hunger, nausea, diarrhoea, keeping aware from emotional and social eating), nil surgical outcomes evaluated | ketonuria and presence of weight loss and weekly monitoring |
| Contreras et al., | BP (decreased), biochemical markers (AST and ALT increased), compliance (94%) to diet and tolerance (high tolerance but some has dizziness, asthenia) with diet + surgical complications (more major complications occurred in LCD compared to VLCD) and hospital stay (no significant differences) | bringing back empty sachets of VLCD and LCD diet, recording non-allowed food items |
|  |  |  |
| Bakker et al., | side effects of diet evaluated through interviews (increased hunger and appetite, diarrhoea, constipation in LCD compared to omega 3), compliance (lower in LCD) was also asked, and 6 month follow up (weight loss continued), no surgical complications measured | Interviews |
| Ministrini et al., | weight loss, liver volume reductions were all secondary measures, HDL, LDL, LFTs were all lower post diet, liver steatosis decreased, no mention of surgical outcomes | ketonuria |
| Pilone et al., | HDL, LDL, LFTs, glucose and insulin all lower, satisfaction with weight loss program evaluated + adverse effects to diet (headaches, cramps, asthenia/weariness, hypotension), no surgical outcomes | ketonuria and plasma ketones |
| Edholm, D et al., | surgical complexity (exposure of the field, liver size, duration of surgery, postoperative complications [minimal- 3 pts anastomotic ulcers], hospital stay [no difference]) | nil description |
| Colles et al., | HDL and LDL, CRP, Hba1c, qualitative measures of side effects and acceptability (taste, nausea [nil reports], vomiting [nil reports]), hunger and emotional eating + social eating increased from week 4 to week 10; constipation, light headedness, cold intolerance, dry skin + surgical outcomes (no major perioperative complications, no open procedures, no prolonged hospital stay) | Ketonuria |
| Ekici and Ferhatoglu | operation duration (reduced) hospital stays (reduced) | Ketonuria |
| Brody, Vaziri, Garey, Shah, LeBrun et al., | ALP, ALT, AST decreased, bilirubin increased, safety, tolerance and acceptability, postoperative complications (GI bleed) | Preoperative liver reduction utilizing a novel nutritional supplement |
| Collins et al., | resolution of co-morbid conditions, postoperative complications (PE, intra-abdominal bleed; no clear decrease), reduction in surgical technical challenge, reduction in operative time, operative blood loss, | taking a history in monitoring and follow up visits |
| Benjaminov et al., | HDL, LDL, TAGs all decreased, ease of access during surgical procedure (subjectively measured to be greater due to reduction in LLL volume to facilitate access to the GOJ + no difficulty in retraction) | Food diary |
| Schiavo, Pilone et al., | biochemical data (LFTs, HDL, LDLs, Cr, vitamin levels), tolerance (hunger, nausea, vomiting, headache, halitosis, and constipation through self-answered questionnaire) | 3-day estimated food records + 72h recalls + total ketone score correlation with weight reduction % |
| Fris | Fat loss no mention of surgical complications | nil mention of evaluation |
| Abidin et al., | ALT but no mention of surgical complications | food diary, |
| Faria et al., | biochemical data (glucose, insulin, CRP, urinalysis, perioperative outcomes of diet i.e., bleeding, presenting of hypertension, access to GOJ, patient reported hunger after diet (no changes), no difference in surgical time between liquid and normal consistency but there was reduction in surgical time overall/cm3 of liver vol loss in liquid diet group | dietary recalls in the beginning, d7 and at the end + ketonuria |
|  |  |  |
| Chakravarty et al., | weight loss and liver volume reductions were secondary measures, surgical time and hospital stay showed no differences | nil description |
| Childs et al., | None other | Didn’t mention |
| Schiavo et al., | Diet compliance scores, diet acceptability and adverse effects to diet. No mention of surgical complications or benefits or perioperative care benefits received form diet | 72-hr recall and 3-day food estimates + urinary ketones |
| Lange et al., | Body fat, visceral fat, Liver function tests, liver histology to determine NAFLD score, operating times and adverse effects due to diet | Food diary |
